# Prevalence of Phantom Scanning in Cardiac Arrest and Trauma Resuscitations: The Scary Truth 

**DOI:** 10.24908/pocus.v8i2.16690

**Published:** 2023-11-27

**Authors:** Zachary Boivin, Curtis Xu, Donias Doko, Meghan Kelly Herbst, Trent She

**Affiliations:** 1 University of Connecticut Emergency Medicine Residency, University of Connecticut School of Medicine Farmington, CT USA; 2 Department of Emergency Medicine, University of Connecticut School of Medicine Farmington, CT USA; 3 Department of Emergency Medicine, Hartford Hospital Hartford, CT USA

**Keywords:** phantom scanning, Trauma, cardiac arrest, ultrasound workflow, point of care ultrasound, POCUS

## Abstract

**Background: **The prevalence of phantom scanning, or point of care ultrasound (POCUS) performed without saving images, has not been well studied. Phantom scanning can negatively affect patient care, reduce billed revenue, and can increase medicolegal liability. We sought to quantify and compare the prevalence of phantom scanning among emergency department (ED) cardiac arrests and trauma resuscitations. **Methods: **This was a single center, retrospective cohort study from July 1, 2019, to July 1, 2021, of all occurrences of POCUS examination documented on the resuscitation run sheet during cardiac arrest and trauma resuscitations. Two investigators reviewed the run sheets to screen for POCUS documentation. Instances where documentation was present were matched with saved images in the picture archiving and communication system. Instances where documentation was present but no images could be located were considered phantom scans. A two-tailed student’s t test was utilized to compare the phantom scanning rate between cardiac arrest and trauma resuscitations. **Results: **A total of 1,862 patients were included in the study period, with 329 cardiac arrests and 401 trauma resuscitations having run sheet documentation of POCUS performance. The phantom scanning rate in cardiac arrests and trauma resuscitations was 70.5% (232/329) and 86.5% (347/401), respectively (p < 0.001). **Conclusion: **Phantom scanning is common in both cardiac arrests and trauma resuscitations in the ED at our institution, but is significantly higher in trauma resuscitations. Further research is needed to assess causes and develop potential solutions to reduce the high prevalence of phantom scanning.

## Background

Performing point of care ultrasound (POCUS) examinations without saving images is both understudied and problematic for emergency departments. Although there is no consensus on the terminology for this practice, it is commonly referred to as phantom scanning 1, 2, 3, 4. Phantom scanning can result in substandard patient care, billing deficiencies, quality assurance and improvement (QA/QI) concerns, and can lead to medicolegal issues. Guidelines from the Centers for Medicare and Medicaid, the American Institute of Ultrasound in Medicine, as well as the American College of Emergency Physicians recommend that all POCUS examinations should include a representative image saved with clinically relevant anatomy and pathology 5, 6. Of note, the American College of Surgeons, which accredits trauma centers, does not have a defined guideline for the performance and saving of Focused Assessment with Sonography for Trauma (FAST) examinations 7.

POCUS plays an important role in cardiac arrest and trauma resuscitations 8, 9. While it is important to consistently save POCUS images, this may not occur for a few reasons. Emergency physicians have time-sensitive responsibilities to care for critically ill patients during resuscitations, which may shift their priorities away from saving images 10, 11, 12. Critically ill patients are often not registered in the electronic medical record (EMR) prior to POCUS examination, which may discourage providers from saving images. The culture of an institution may be more focused on performing POCUS and less focused on the saving of POCUS images. Physicians may not realize the benefit of saving images or may think that if no images are saved, then further documentation is not needed.

There is a lack of research on the underlying causes of phantom scanning in the emergency department (ED), even though it is commonly acknowledged in POCUS studies 3, 13, 14. Previous studies have shown that improving POCUS workflow or offering physician incentives have led to increased compliance with POCUS documentation, but none have looked at their effect on phantom scanning 2, 15, 16, 17. Our aims were to determine the prevalence of phantom scanning among cardiac arrests and trauma resuscitations and compare these two groups. We focused on cardiac arrests and trauma resuscitations because comprehensive documentation of events is recorded in real time by a dedicated ED nurse in both settings, and the need for rapid and timely POCUS performance makes these resuscitations susceptible to phantom scanning. Our a priori hypothesis was that the phantom scanning prevalence would be higher among cardiac arrest than trauma resuscitations because the FAST examination is an adjunct to the trauma primary survey, whereas POCUS during cardiac arrest is not as broadly or formally recommended 18, 19. 

## Methods

### Study Design

This was a single center, retrospective cohort study between July 1, 2019, and July 1, 2021. Inclusion criteria were ED patients ≥18 years old who arrived as a trauma or cardiac arrest resuscitation. Patients were excluded if they did not have a POCUS documented by a nurse in the resuscitation run sheet, or if the timestamp of POCUS performance on the run sheet occurred after return of spontaneous circulation (ROSC) among cardiac arrest patients, as the time-sensitive nature of a cardiac arrest POCUS is no longer present. A trauma resuscitation was defined as a patient with a life-threatening traumatic injury or meeting specific trauma criteria defined by the institution (Table 1), and a cardiac arrest resuscitation was defined as loss of pulses requiring initiation of Advanced Cardiac Life Support. In both cases, a critical care trained ED nurse was present and dedicated to documenting on an electronic run sheet in real time all patient interventions such as medications administered, procedures, and POCUS performance with timestamps. During documentation, our nurses are also trained to ask about POCUS findings if they are not explicitly mentioned by providers during the resuscitation. As each resuscitation always had one nurse assigned to documentation and because each resuscitation had an electronic run sheet recorded, this run sheet documentation was used as a surrogate for a POCUS examination occurring during the resuscitation. 

**Table 1:** Trauma Activation Criteria

· Glasgow Coma Scale £ 12
· Systolic Blood Pressure < 90 mmHg
· Respiratory rate < 10 or > 29, intubated or with threatened airway
· Any penetrating injury to the head, neck, torso, or extremities proximal to the elbows or knees
· Motor Vehicle Collision with patient ejected from vehicle
· Fall > 20 feet
· Major deep burns (20% body surface area)
· Chest wall instability
· Two or more proximal long bone fractures
· Suspected unstable pelvic fracture
· Open or depressed skull fracture
· Paralysis secondary to injury
· Amputation proximal to wrist or ankle
· Crushed, degloved, or mangled extremity at or proximal to elbows or knees
· Pregnant patient 20 weeks or greater with vaginal bleeding, abdominal tenderness, and/or injury
· Transfer patients receiving blood to maintain vital signs, positive Focused Assessment with Sonography for Trauma examination, or chest tube insertion
· Emergency Department Physician’s discretion

### Setting

The study was performed at a Level 1 trauma center with over 110,000 annual patient visit, and an active ED POCUS program with approximately 4,500 POCUS examinations performed annually. The ED is staffed by 48 emergency medicine attendings, 54 rotating emergency medicine residents, and five emergency medicine fellows (one ultrasound, two resuscitation, and two simulation fellows). Residents participate in 16 hours of ultrasound training during their residency orientation, spend four weeks on a dedicated POCUS rotation, and must perform a minimum of 500 POCUS examinations as a requirement for graduation.

There is a six-bed critical care area where resuscitations take place, including trauma and cardiac arrest resuscitations, with a dedicated Sonosite X-porte (FUJIFILM Sonosite, Inc., Bothell, WA) ultrasound machine. This area also has a dedicated three-person nursing team as well as a care team consisting of an attending emergency medicine physician and frequently a resident, fellow, or both. POCUS examinations are primarily performed by an emergency medicine resident with supervision by an emergency medicine attending, unless there are no emergency medicine residents in the department, which occurs during weekly resident conference and occasional residency events. During these rare occasions, FAST examinations are performed by a member of the trauma team (an advanced practice provider, surgery resident, trauma fellow, or the attending trauma surgeon) with supervision by the emergency medicine attending.

Our institution’s current POCUS workflow is order-based. First, an order needs to be placed by an emergency medicine provider in the EMR, then the patient information is queried on the ultrasound machine and the correct patient is selected. Images are acquired and saved by an operator, and when the examination is ended on the machine, they upload to the picture archival and communication system (PACS). Emergency physicians document their findings in the EMR, which is linked to the images stored in the PACS. If POCUS images are saved without patient information, the images are still uploaded to the PACS and available for viewing under an anonymous designation, and can later be merged with the patient’s chart in the EMR. 

### Chart Review

Patients with cardiac arrest were determined by ICD-10 coding in the EMR. Trauma resuscitations were identified utilizing a pre-existing trauma registry in the EMR documenting all trauma team activations. Manual chart review and abstraction was completed by two investigators (ZB, CX) unblinded to the research question, and the principal investigator (TS) randomly selected 10% of cases to review for accuracy. Study investigators met prior to chart review to develop a systematic review process for both the chart and the ultrasound image review process.

A phantom scan was defined as a POCUS scan that was performed on a patient, where no subsequent images were saved. Performance of a POCUS was defined as any POCUS notation that was documented on the resuscitation run sheet by a nurse. Images were considered saved if an examination was located on our PACS that could be corroborated with the patient.

Specifically, for patients who had either a cardiac arrest or trauma resuscitation, the study investigators first determined whether there was a POCUS examination documented in the resuscitation run sheet. If documentation stated a POCUS was performed, the investigators then reviewed the PACS for POCUS images that corresponded with the patient according to timestamp, examination type, and POCUS findings. If there were no images that matched the run sheet documentation, the POCUS examination was categorized as a phantom scan. Investigators only evaluated the initial POCUS examination performed in a resuscitation; subsequent POCUS examinations were not evaluated. 

To ensure the resuscitation run sheet was accurate in documenting the performance of a POCUS examination, the investigators reviewed the PACS system for 10% of all resuscitations, both trauma and cardiac arrest, where there was no documented POCUS on the resuscitation run sheet for images in our PACS. This was done to ensure that our screening method of using the resuscitation sheet for a performed POCUS was accurate. 

### Outcomes

The primary outcome for the study was the prevalence of phantom scanning, which was calculated by dividing the number of patients with POCUS images linked to the patient encounter by the total number of patients with a POCUS examination documented in the resuscitation run sheet. The secondary outcome was the comparison of phantom scanning between cardiac arrest and trauma resuscitations.

### Data Analysis

The prevalence of phantom scanning was calculated in Microsoft Excel (Redmond, WA). A two-tailed Mann-Whitney U test using IBM SPSS version 27 (Armonk, NY) was performed to assess for a difference between phantom scanning in cardiac arrest and trauma resuscitations, with alpha <0.05 indicating significance. 

## Results

**Figure 1:**
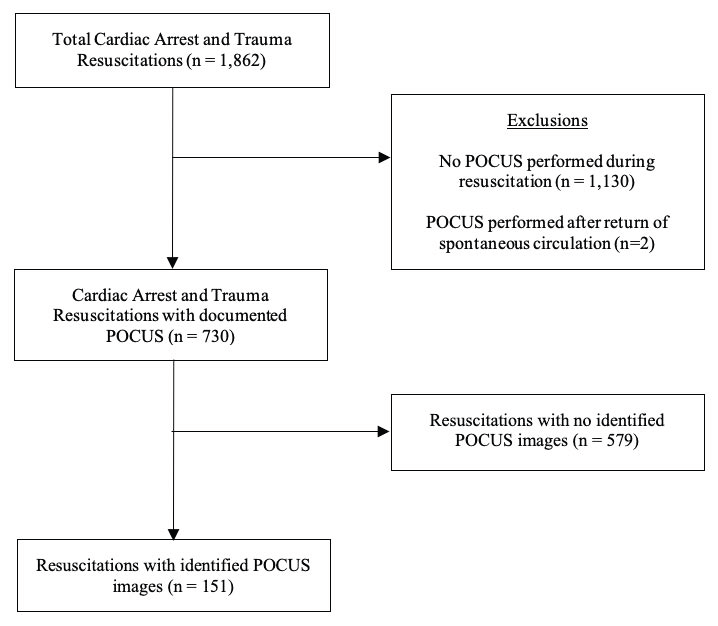
Flowchart describing inclusion and exclusion criteria.

We reviewed 1,862 patient resuscitations for this study: 861 cardiac arrests and 1001 trauma resuscitations. A total of 1,130 patients were excluded due to a lack of POCUS documentation in the run sheet, and two patients were excluded for a POCUS performed after ROSC was obtained, leaving 730 patients (329 cardiac arrests and 401 trauma resuscitations) for analysis (Figure 1). To test for accuracy of resuscitation run sheet documentation for POCUS performance, we looked at a randomized 10% selection of the previously excluded resuscitations due to a lack of POCUS documentation on the resuscitation run sheet. The PACS was reviewed for these patients and found POCUS was performed for 1/60 (1.7%) of traumas and 0/50 (0%) of cardiac arrest resuscitations. 

A total of 151 patients had identified POCUS images in the PACS. For cardiac arrests, POCUS images were identified in 29.5% of included cases (n=97) resulting in a 70.5% phantom scanning rate among cases with POCUS documented as performed. In trauma resuscitations, POCUS images were identified in 13.5% of included cases (n=54), resulting in an 86.5% phantom scanning rate among cases with POCUS documented as performed. The prevalence of phantom scanning was significantly higher in trauma resuscitations compared to cardiac arrest resuscitations (p<0.001).

## Discussion

To our knowledge, this is the first study to quantify the rate of phantom POCUS scanning, however, this study took place at a single institution, which limits its generalizability. While phantom scanning is a known problem in the POCUS field, the extent to which it occurs has not been well studied. Our results support that phantom scanning during resuscitations is common at our institution, which raises several concerns for our institution and other EDs with similar POCUS workflows. First, despite being able to readily utilize POCUS at the bedside, decreased adherence to emergency medicine societal guidelines for image retention and documentation could negatively impact patient care 3, 6. Second, phantom scanning exposes physicians to QA/QI concerns, as providers are unable to obtain feedback on their acquisition and interpretation of POCUS images, and thus are unable to improve. Third, phantom scanning presents several medicolegal issues 3. This issue is apparent when considering that POCUS, especially in the setting of critically ill patients, provides vital information for clinical decision-making when other tests or evaluations are not yet available or are nondiagnostic 20. When major decisions to transport a patient to the OR, cease resuscitation, or perform procedures such as a thoracotomy or pericardiocentesis are based on POCUS, it is paramount that evidence of the legitimacy of those decisions are readily available for future case review. Without images available for review, medical decisions may appear unwarranted. Finally, phantom scanning eliminates the potential to bill for the POCUS examination 5. As POCUS use increases in the ED, the number of consulting and admitting providers who review images obtained by emergency physicians to direct patient care will continue to increase 21. As more robust POCUS programs expand from academic emergency medicine into the community setting, limiting the amount of phantom scanning will ensure continued growth of POCUS within the specialty of emergency medicine 22. 

When comparing the rate of phantom scanning in trauma resuscitations to cardiac arrest, our initial hypothesis that there would be a lower phantom scanning rate for the FAST examination was incorrect. This finding caused us to give further thought as to why the FAST examination phantom scanning rate was significantly higher than in cardiac arrest resuscitations. While the trauma team at our institution only occasionally performs the FAST examination, their involvement poses several issues pertaining to POCUS phantom scanning rate. The trauma team receives no formal education on the ED POCUS workflow and the need to save images. Additionally, the trauma providers use a different set of ultrasound machines when not in the ED and so may be unfamiliar with the ED ultrasound machines and the process of saving images on them. Finally, members of the trauma team are unable to access the POCUS ordering system as the system is only available for ED providers, and the lack of an order may deter the provider performing the FAST from saving images. The FAST examination may take longer to acquire as there are four standard views instead of a minimum two views (oftentimes one view) for cardiac arrest POCUS, there is no dedicated pause in care for the FAST examination as there is in cardiac arrest pulse checks, and the FAST examination may be interrupted to take the patient for a computed tomography examination or to the operating room. The differences between how POCUS is performed during a cardiac arrest and trauma resuscitation could have caused the difference in phantom scanning rate, but does not change the overall high phantom scanning rate at our institution for these resuscitations. 

We propose the following interventions to decrease phantom scanning rates at institutions with similar processes. First, formal education to avoid phantom scanning presented to both emergency medicine physicians and consultants who perform POCUS examinations in the ED. While emergency physicians are aware of the POCUS workflow, educating consultants may help reduce the rate of phantom scanning by providing insight into the POCUS workflow and increasing familiarity with the ED POCUS machines, which is recommended in the American College of Emergency Physicians ultrasound guidelines 6. Second, scheduled reminders to physicians about saving images should be made at regular intervals. Finally, simple interventions such as labeling the ultrasound machine in the resuscitation area reminding physicians to order and save POCUS images, or saving unordered examinations under a common name such as “Code” or “Trauma” to assist with image review and QA/QI. These interventions, and their effect on the prevalence of phantom scanning, offer an opportunity for further research to help minimize phantom scanning and to optimize patient care. 

### Limitations

The major limitation of this study was that it only looks at a single POCUS workflow at a single institution, making it difficult to generalize our conclusions to other departments. Other institutions workflow may differ in how orders are placed, the type of workflow, or who performs the FAST examination. We hope, however, this study provides a replicable methodology that encourages other institutions to review their own POCUS workflows, assess their own rates of phantom scanning, and—most importantly—encourage their physicians to save their obtained POCUS images. An additional limitation was its retrospective nature, which meant we were unable to definitively establish any causal relationships as to why there was such a high prevalence of phantom scanning at our institution. We also had to use resuscitation run sheet documentation as a surrogate for POCUS performance rather than direct visualization or acknowledgment of POCUS performance. It is possible there were other cardiac arrest or trauma resuscitations where POCUS was utilized but was not documented in the run sheet, but our review of 10% of randomly selected resuscitations where POCUS was not documented in the run sheet found less than one percent (1/110) discrepancy, suggesting our surrogate marker for POCUS performance was accurate. Additionally, there could have been POCUS images saved under a different patient name if the previous POCUS examination was not ended, or the images could have been saved but failed to upload to the PACS, but we attempted to mitigate this by searching by time performed in the PACS, allowing investigators to review all POCUS examinations conducted at approximately the time as the resuscitation. 

## Conclusion

At our institution, we found a 70.5% phantom scanning prevalence during cardiac arrest resuscitations, and an 86.5% phantom scanning prevalence for trauma resuscitations. Trauma resuscitations had a significantly higher phantom scanning prevalence and lower number of formal POCUS orders when compared to cardiac arrest resuscitations. 

## Statement of Ethics

IRB approval was obtained for this study.

## Disclosures

The authors report no disclosures related to this work.
